# Insights into the piezo-photocatalytic activity and optimized magnetic recovery of hybrid bismuth ferrite-based nanosystems

**DOI:** 10.1039/d5na00646e

**Published:** 2025-08-26

**Authors:** P. Maltoni, N. Ghibaudo, A. Kumar, G. Barucca, M. Vocciante, F. Locardi, G. Varvaro, S. Slimani, M. Ferretti, T. Sarkar, A. Reverberi, S. Alberti, D. Peddis

**Affiliations:** a Department of Chemistry and Industrial Chemistry & INSTM RU, University of Genoa Via Dodecaneso 31 16146 Genova (GE) Italy pierfrancesco.maltoni@unige.it stefano.alberti@unige.it; b Institute of Structure of Matter (ISM), National Research Council (CNR), nM2-Lab Via Salaria km 29.300, Monterotondo Scalo Roma 00015 Italy; c Department of Science and Engineering of Matter, Environment and Urban Planning (SIMAU), University Politecnica delle Marche Via Brecce Bianche 12 60131 Ancona Italy; d Department of Materials Science and Engineering, Uppsala University Box 35 SE 75103 Uppsala Sweden

## Abstract

Bismuth ferrite (BiFeO_3_), a perovskite oxide with both ferroelectric and antiferromagnetic properties, has emerged as a promising material for environmental cleanup due to its piezo-photocatalytic activity. The material's ability to degrade organic pollutants, such as azo dyes, under both light irradiation and mechanical stress (ultrasonic waves) offers a dual-action mechanism for efficient wastewater treatment. In this work, we explore the synthesis of BiFeO_3_ nanoparticles *via* a simple sol–gel method, followed by characterization of their structural, magnetic, and photocatalytic properties. Under ultrasonic treatment, BiFeO_3_ generates piezoelectric potentials that enhance electron–hole separation, promoting photocatalytic degradation of methylene blue. The combination of photocatalysis and piezocatalysis improves catalytic efficiency while reducing energy consumption compared to traditional UV-based photocatalysis. Additionally, coupling BiFeO_3_ with cobalt ferrite (CoFe_2_O_4_) creates a magnetically recoverable system, facilitating efficient catalyst separation from treated water, and modifying the kinetic process of photodissociation. The magnetic recovery was improved through the development of a tailored magnetic support system, designed to optimize the spatial magnetic field gradient. These findings highlight the potential of BiFeO_3_-based nanosystems for sustainable, energy-efficient, and eco-friendly solutions to water pollution, addressing both dye degradation and the need for effective water remediation techniques.

## Abstract 2

BiFeO_3_ nanoparticles, combined with CoFe_2_O_4_ for magnetic recovery, show piezo-photocatalytic degradation of dyes under visible light and ultrasound, offering an energy-efficient route for water remediation.

## Graphical abstract

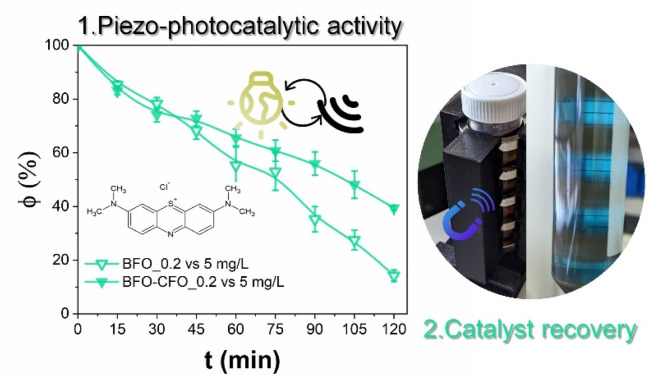

## Introduction

Multifunctional nanostructured materials that integrate both catalytic and magnetic properties have recently emerged as a promising strategy for environmental remediation, by combining the catalytic functionality with magnetic responsiveness, the latter enabling efficient contaminant removal and catalyst recovery under an external magnetic field.^1–6^ Bismuth ferrite (BiFeO_3_, BFO), a visible-light-responsive perovskite oxide, has gained attention due to its ability to degrade a wide range of organic pollutants, including persistent azo dyes, under light irradiation.^7^ Its effectiveness is further enhanced by nanostructuring, and modifications such as doping or the creation of heterojunctions, which improve charge separation efficiency.^8,9^ To overcome the limitations of traditional wastewater treatments—such as high sludge production and limited degradation capacity—photocatalytic (light plus catalyst) and piezocatalytic (ultrasound plus catalyst) technologies offer a sustainable solution to waste water pollution.^10^ BFO offers several advantages as a photocatalyst for organic dye photodissociation. With the relatively low bandgap (∼2–2.7 eV),^11,12^ it can be activated under ambient sunlight, making it a promising candidate for sustainable water treatment. Furthermore, the piezoelectric properties, enabling piezocatalysis under mechanical stress (*e.g.*, ultrasound), may enhance the catalytic activity even in the absence of light, thus broadening the operation conditions.^13^ BFO crystallize in a rhombohedrally distorted perovskite structure (space group *R*3*c*), which enables their multiferroic character.^14^ The ferroelectricity originates from the stereochemically active 6s^2^ lone pair of Bi^3+^ ions and the cooperative displacement of both Bi^3+^ and Fe^3+^ along the [111] direction, leading to a strong spontaneous polarization (up to ∼90 μC cm^−2^ in single crystals).^15^ This ferroelectric order remains stable up to the Curie temperature (*T*_C_ ≈ 1100 K), where the material transits to a paraelectric phase.^16^ On the magnetic side, BFO exhibits G-type antiferromagnetic ordering below its Néel temperature (*T*_N_ ≈ 700 K), where Fe^3+^ spins are antiparallel.^16,17^ However, due to a slight canting of these spins from the Dzyaloshinskii–Moriya interaction, a weak net magnetization may appear at room temperature, especially enhanced at the nanoscale due to uncompensated spins and surface effects.^18^

The improvement of photocatalytic activity has been a focal point of numerous studies.^19^ For instance, it was demonstrated that integrating BFO nanoparticles supported on polymers enhances photocatalytic performance under visible light irradiation.^20^ Similarly, Li *et al.* explored the photodissociation of organic pollutants by manipulating the polarization of BFO-based materials, leading to improved activity.^21^ The structural and compositional modifications have also been investigated to optimize its properties, revealing that specific dopants can significantly influence its behavior.^22^ Additionally, Mejía Gómez *et al.* conducted structural studies on yttrium substituted BFO, providing insights into the material's stability and functional properties.^23^ When comparing the energy demands of conventional methods to BFO-based photocatalysis, the energy consumption for photocatalytic degradation using BFO under visible light is significantly lower than that of UV-based photocatalysis (up to 400 W), which requires high-energy UV lamps. Moreover, when paired with piezocatalysis under mechanical energy (*e.g.*, 120 W ultrasonic bath), the process can become even more energy-efficient.^24^ Interestingly, when aqueous dispersions of nanocrystals are subjected to ultrasonic waves (typically 20–40 kHz), cavitation phenomena occur in the surrounding liquid.^25^ The collapse of cavitation bubbles creates localized hotspots with estimated transient temperatures of ∼1000–2000 K and pressures of up to ∼100 atm.^26,27^ These conditions generate strong mechanical stress on the nanoparticle surfaces. For piezoelectric BFO, this stress can induce temporary charge separation (piezoelectric potential),^28^ which can assist in separating photogenerated electron–hole pairs across its bandgap.^11,12^ This hybrid mechanism—known as piezo-photocatalysis (light plus ultrasound plus catalyst)—can significantly enhance catalytic activity under light and mechanical stimuli,^29–31^ enabling applications such as dye degradation.^32,33^ Furthermore, it offers significant advantages in terms of sustainability and environmental impact, as it utilizes renewable energy sources (light and mechanical vibrations), avoids toxic chemicals, and ensures high recyclability of the catalyst.^34,35^ Despite these advancements,^36–38^ challenges remain in fully harnessing the potential of BFO as a piezo-photocatalytic material.^39^

One of the most critical limitations in photocatalysis lies in the recovery and reuse of nanocatalysts after treatment. Many conventional photocatalysts,^40–42^ such as TiO_2_, Cu_2_O, and CdS, are non-magnetic, while others like nanostructured BFO are antiferromagnetic or only weakly ferromagnetic. As a result, they cannot be effectively separated from water using an external magnetic field, making post-treatment recovery challenging and raising concerns about secondary contamination and limited reusability. To overcome this, BFO can be coupled with ferro(i)magnetic nanoparticles (in this case CoFe_2_O_4_, CFO), to develop a magnetically recoverable catalyst system. This allows easy separation of the nanoparticles from the treated water by applying an external magnetic field,^43,44^ minimizing secondary contamination and improving reusability.^45^ Such systems leverage the piezo-photocatalytic properties of BFO and the magnetic handling capabilities of CFO, paving the way for practical applications in water purification and environmental cleanup, yet to be achieved with classical photocatalysts.^46^ To the best of our knowledge, however, the design and optimization of a system that fully exploits both the piezo-photocatalytic activity of BFO and the magnetic recoverability conferred by CFO remains underexplored.

This study focuses on the piezo-photocalytic effects of BFO–CFO nanocrystals on the degradation of two organic dyes (methylene blue, MB, and methyl orange, MO), which is estimated by UV-VIS spectroscopy. These results established a protocol to maximise the photodissociation of MB specifically, and, subsequently, to design a multifunctional system in which the coupling with a magnetic phase (CFO) enabled the recovery and reuse of the hybrid BFO–CFO system from the water suspension. Kinetic modeling of MB degradation revealed a shift from first-order to fractional-order behavior (*α* = 0.29) upon CFO coupling, highlighting the influence of surface heterogeneity on photocatalytic efficiency. The custom magnetic separator based on ring-shaped NdFeB permanent magnets was designed, and the magnets configuration optimised to maximize magnetic field gradients for rapid and effective catalyst separation under static conditions.

## Results and discussion

**Fig. 1:**
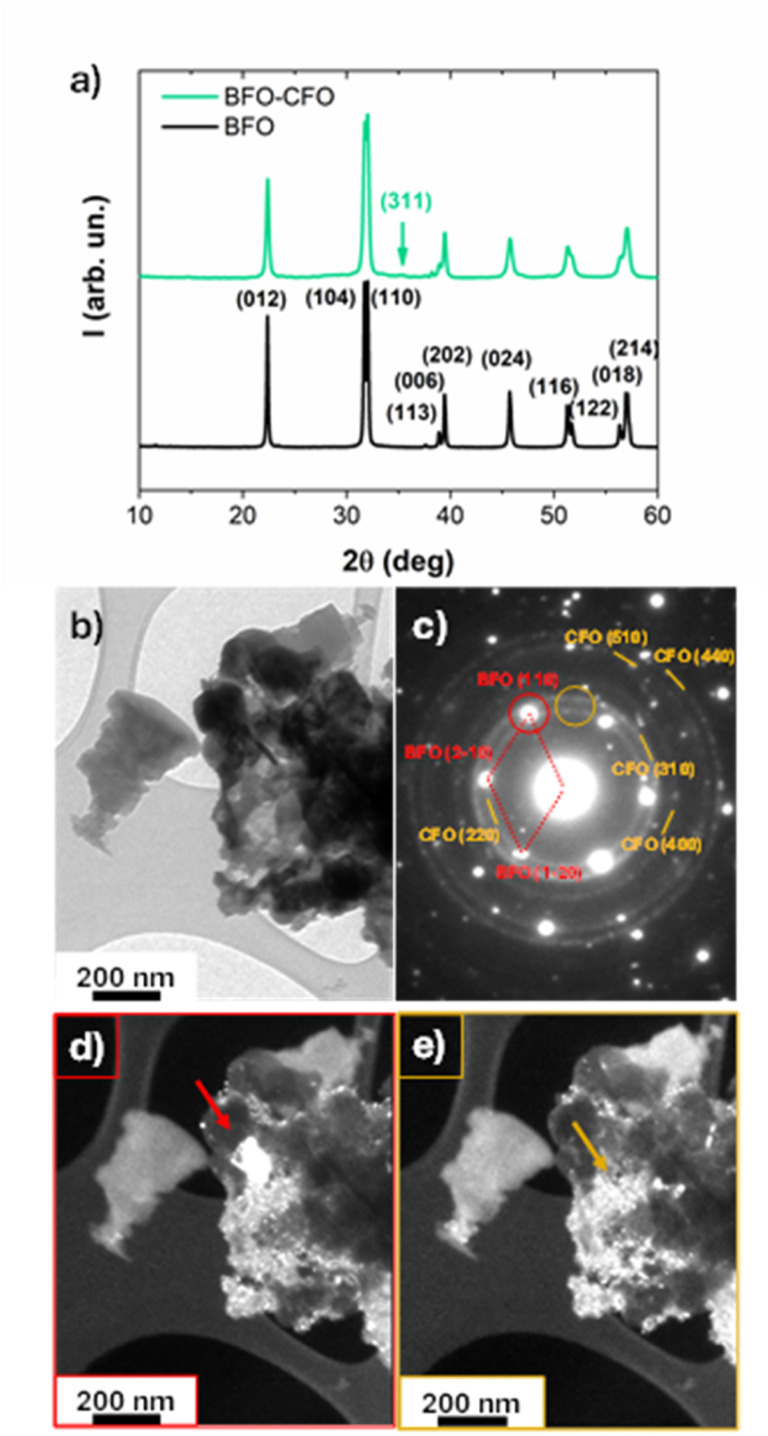
(a) XRPD patterns of pure BFO and BFO–CFO nanocomposite; (b–e) bright-field TEM images of BFO–CFO: (b) general view; (c) corresponding SAED pattern: diffraction rings are attributed to the CFO phase, while the diffraction spots correspond to the BFO phase (the red cell refers to a BFO grain in [001] zone axis); (d) dark-field image obtained from the diffraction spots in the red circle: a large grain of BFO appears whiter (red arrow); (e) dark-field image obtained from the diffraction spots in the orange circle: the small CFO grains appear whiter (orange arrow).

In the broad framework of BFO/CFO nanocomposites, we synthesized a hybrid system by adapting the procedure reported by Sarkar *et al.*^47^ This approach enabled the formation of a heterostructure combining the piezoelectric properties of BFO with the magnetic characteristics of CFO. The resulting material was characterized to evaluate its structural, morphological, and functional properties, with a focus on its potential for magnetically recoverable piezo-photocatalytic applications. The structural features of BFO-based samples were examined using X-ray powder diffraction (XRPD) and transmission electron microscopy (TEM), as shown in Fig. 1. The diffraction patterns of the pristine BFO sample (Fig. 1a) display reflections characteristic of the rhombohedral perovskite structure, consistent with the *R*3*c* space group.^48^ In the case of BFO–CFO nanocomposite containing 10 at% of the Co-ferrite phase by atomic ratio (verified by EDS analysis, see Fig. S1), the XRD diffraction peaks corresponding to the CFO spinel phase (*Fd*3̄*m*) are either not observable or only weak reflections, such as the (311) peak, are detected. This is expected due to the relatively low content of the magnetic phase compared to the dominant BFO matrix. Notably, no additional reflections indicative of secondary or impurity phases are visible, indicating phase-pure systems. The average crystallite sizes of BFO, calculated *via* Scherrer equation, corresponds to ∼25 nm. It is worth noting that achieving phase pure BFO required careful tuning of the synthesis parameters. This approach enabled the stabilization of stoichiometric nanostructured BFO even after annealing at moderate temperatures (500 °C). EDS analysis performed on BFO and BFO–CFO composite confirmed the intended composition within experimental uncertainty (see Fig. S1 in SI), validating the synthesis protocol.

**Fig. 2:**
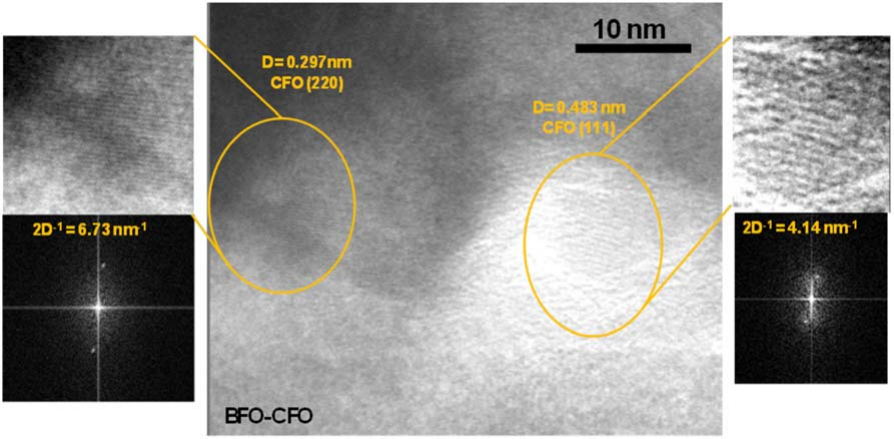
HR-TEM pictures of BFO–CFO composite; the interplanar spacings of 0.297 nm and 0.483 nm, obtained through the FFT of the image and corresponding to the (220) and (111) planes of the spinel CFO phase, are shown in the panel together with the FFTs and magnified details of the atomic planes.

TEM analysis was performed to investigate the inner morphology of the samples. Bright-field TEM images of BFO (Fig. S2 in SI) reveal large agglomerates composed of densely packed nanocrystals forming interconnected polycrystalline structures with overall aggregate sizes ranging from a few hundred nanometres up to microns. Selected-area electron diffraction (SAED) patterns obtained from these agglomerates confirm the crystalline nature of the powders, and all the interplanar distances were ascribable to the rhombohedral BFO phase (International Centre for Diffraction Data (ICDD) card no 71-2494). Regarding the composite (Fig. 1b), the more intense diffraction spots were associated with the BFO phase, while feeble spots, arranged in circles, are attributed to the CFO spinel structure (Fig. 1c). This result indicates that CFO grains have a smaller size compared to the BFO ones, rendering their reflections poorly visible in the XRD patterns. To resolve the spatial distribution of these two phases, dark-field imaging was performed. When the image was formed using the BFO-related diffraction spots (red circle), a large, bright BFO grain became visible (red arrow in Fig. 1d), accompanied by several smaller, less intense CFO grains whose diffraction spots cannot be removed by the finite dimension of the diaphragm. In contrast, the dark-field image obtained using the CFO-related diffraction spots (orange circle) reveals enhanced contrast only from the smaller CFO grains, which appear brighter and uniformly dispersed (orange arrow in Fig. 1e). This imaging approach, performed in different areas of the sample, has allowed confirming the coexistence of distinct grain sizes and crystallinities for the two phases, with BFO forming larger domains and CFO remaining finely dispersed. High-resolution transmission electron microscopy (HR-TEM) was employed to further investigate the structural features of CFO grains embedded within the BFO matrix (Fig. 2). The analysis reveals well-defined crystalline domains of ∼10 nm corresponding to the CFO phase, distinguishable by their lattice fringes. Fast Fourier Transform (FFT) analysis of the HR-TEM images enables the identification of specific interplanar spacings (*D*-values) associated with the spinel structure of CFO. Two distinct lattice spacings were observed in Fig. 2: an interplanar distance *D* = 0.297 nm, which corresponds to the (220) crystallographic plane of CFO, and *D* = 0.483 nm, attributed to the (111) plane of the CFO spinel structure. These values are consistent with the cubic inverse spinel structure of CoFe_2_O_4_ and confirm the presence of small well-crystallized CFO grains within the BFO matrix. The coexistence of different atomic planes in the HR-TEM image and, more generally, of many diffraction spots circularly distributed in the SAED patterns indicates that the CFO nanoparticles are randomly oriented within the composite system, supporting a heterogeneous microstructural integration between the ferrite phases.

**Fig. 3:**
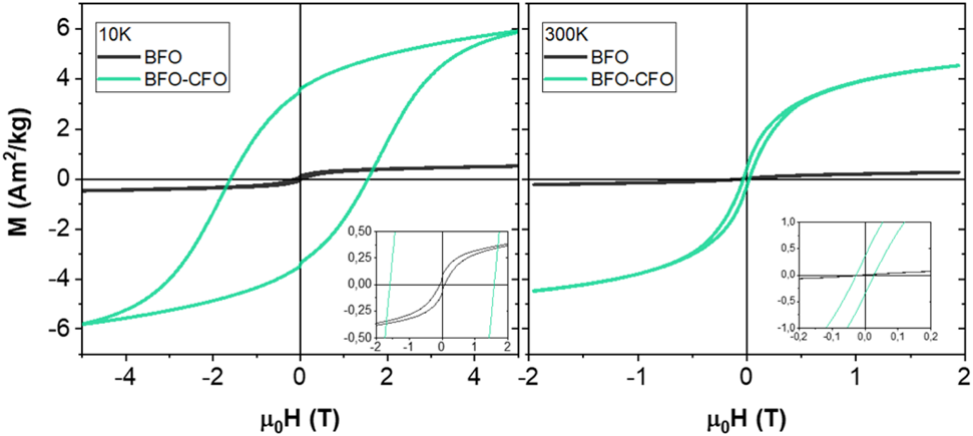
M(H) loops at 10 K and 300 K for BFO and BFO–CFO nanosystems.

From a magnetic point of view, BFO displays antiferromagnetic characteristics, resulting in a magnetization at 10 K that remains unsaturated even at high applied magnetic fields, reaching ∼0.5 Am^2^ kg^−1^ at 5 T (Fig. 3) The observed remanent magnetization and coercivity, although small, are indicative of a weak ferromagnetic component, likely originating from finite-size effects.^49^ BFO–CFO nanocomposite exhibits a uniform magnetic response at 10 K (*i.e.*, a single hysteresis loop), underscoring the strong interfacial coupling between the two materials, with a coercivity of ∼1.58 T being the overall magnetic behaviour dominated by the CFO phase.^50^ At 300 K the system retains a finite magnetization despite a significant reduction in remanence and coercivity due to the CFO nanoparticles approaching the superparamagnetic size threshold,^51–53^ thereby enabling efficient magnetic separation and recovery of the catalyst from the reaction environment. Such magnetically retrievable systems are particularly advantageous in heterogeneous catalysis,^54,55^ where reusability and separation are critical for sustainable applications.^56^

### BFO nanoparticles: photocatalytic and piezo-photocatalytic properties

The photocatalytic (light and catalyst) activity of BFO nanocrystals was first evaluated under simulated solar irradiation using a standard degradation test of methylene blue (MB, 10 mg L^−1^), with BFO loaded at 0.5 g L^−1^. Specifically, all experiments were performed in 100 mL borosilicate glass beaker and a magnetic stirrer was employed to ensure homogeneity during the reaction. As shown in Fig. S3, BFO exhibits negligible photocatalytic activity compared to the benchmark TiO_2_ (Degussa P25), which shows significantly higher photodissociation efficiency, indicating the poor performance of BFO under sole light irradiation (residual methylene blue (MB) fraction (*φ*) ∼90%). This limited activity can be attributed to a combination of fast recombination of photogenerated electron–hole pairs and the relatively low conduction band potential of BFO. Based on the measured optical bandgap (∼2 eV, Fig. S4) from UV-VIS spectrum,^57,58^ and the known electronegativity of BFO, the estimated conduction band edge lies around +0.4 eV—too low to drive the reduction of molecular oxygen, thus limiting reactive oxygen species (ROS) generation crucial for dye degradation.^59^ These ROS are primarily responsible for attacking and breaking down the organic dye molecules in aqueous media.

**Fig. 4:**
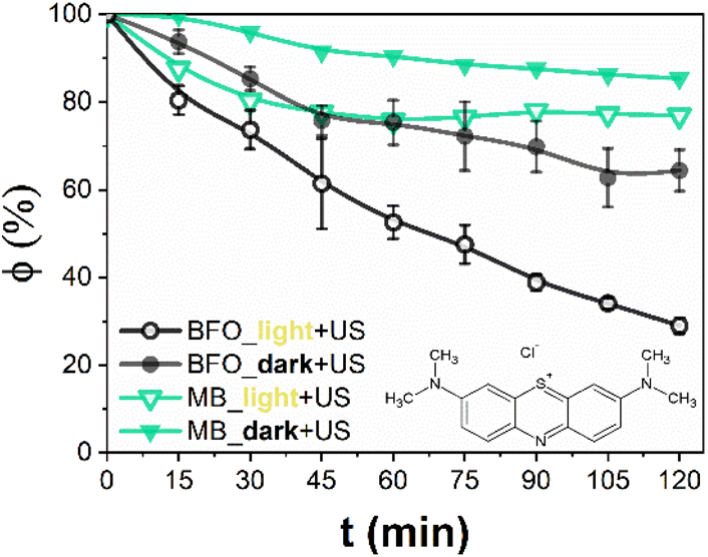
Residual methylene blue (MB) fraction (*φ*) after 120 min, obtained from piezo-photocatalytic (light on and ultrasounds) and piezo-catalytic (light off, dark, and ultrasounds) experiments (0.5 g L^−1^ load of BFO *vs.* 10 mg L^−1^ of MB).

To overcome the poor performance of BFO under sole light irradiation, piezocatalytic (ultrasound and catalyst) and piezo-photocatalytic (ultrasound, light, and catalyst) experiments were conducted by introducing ultrasonic vibration (using an ultrasonic bath, without magnetic stirring), either alone or combined with solar light (Fig. 4). The combination of mechanical stimulation from the ultrasonic bath and photo-excitation from a 300 W Xenon lamp creates a synergistic effect known as piezo-photocatalysis, which has been demonstrated to boost the efficiency of redox reactions such as dye degradation.^60^ Mechanical stress from ultrasonic cavitation activates the piezoelectric domains of BFO, creating transient electric fields that help separate electron–hole pairs more efficiently, consequently promoting the formation of ROS even in the absence of light. To estimate the magnitude of the piezoelectric effect in BFO nanoparticles during ultrasonic treatment, we consider the stress-induced piezoelectric potential generated by cavitation in an ultrasonic bath.^61^ When a dispersion of nanoparticles is exposed to ultrasonic waves (in our case, 35 kHz, 120 W), rapid bubble formation and collapse (acoustic cavitation) in the surrounding liquid generates localized mechanical stress.^62,63^ These stresses can reach pressures in the range of approximately ∼100 atm.^64^ For a piezoelectric material like BFO, such stress can induce a surface potential *via* the direct piezoelectric effect.^37^ The induced piezoelectric voltage (V) across a nanoparticle can be estimated using the following relation:^65^*V* = *d*_33_·*σ*·*t*/*ε*_0_*ε*_r_*V*, where *d*_33_ is the longitudinal piezoelectric coefficient, *σ* is the applied stress, *t* is the characteristic size of the nanoparticle along the stress axis, *ε*_0_ is the vacuum permittivity, and *ε*_r_ is the relative permittivity of BFO. This suggests that a single BFO nanoparticle under sono-mechanical stress could generate a piezoelectric potential of the order of ∼1–2 V. This voltage is significant enough to promote the separation of photogenerated electron–hole pairs, enhancing the catalytic activity of the material. Indeed, the inclusion of BFO led to a clear enhancement of degradation efficiency, particularly under piezo-photocatalytic conditions, where the synergistic effect of mechanical and light energy enabled a degradation yield of approximately 72% after 120 minutes (compared to ∼20% in the dark). This effect is ascribed to the catalyst only, since control experiments confirmed that neither sonolysis (ultrasound, no catalyst) nor photolysis (light, no catalyst) alone significantly drove the degradation of MB in the absence of the catalyst.

Encouraged by these results, the degradation of methyl orange (MO), an anionic dye, was also explored under similar experimental conditions (Fig. S5). In this case, however, the degradation curves under light and ultrasound showed minimal differentiation, suggesting limited or non-specific interaction between BFO and the MO molecule under the tested conditions. This lower activity may stem from MO's molecular structure and its charge state at neutral pH. MO, being an anionic dye, likely experiences repulsion from the negatively charged BFO surface (at near-neutral pH), reducing adsorption and thus limiting ROS-mediated attack. In contrast, MB is a cationic dye, which more readily interacts with the catalyst surface *via* electrostatic attraction, enhancing the probability of degradation by photogenerated radicals. Furthermore, the molecular structure of both dyes results in two values of molar absorption coefficients, which are reported in the literature to be 14 650 L mol^−1^ cm^−1^ and 67 100 L mol^−1^ cm^−1^ for MO and MB, respectively.^66,67^ For this reason, even small variations in the dye concentration result in a much more marked change in absorbance for MB than for MO. Eventually, MO has an azo (–N=N–) group that is known to be more resistant to oxidative attack than the aromatic structures in MB, which may also contribute to its lower degradation rates under the same experimental conditions.

### BFO–CFO nanocomposites for piezo-photocatalysis: properties and kinetics

**Fig. 5:**
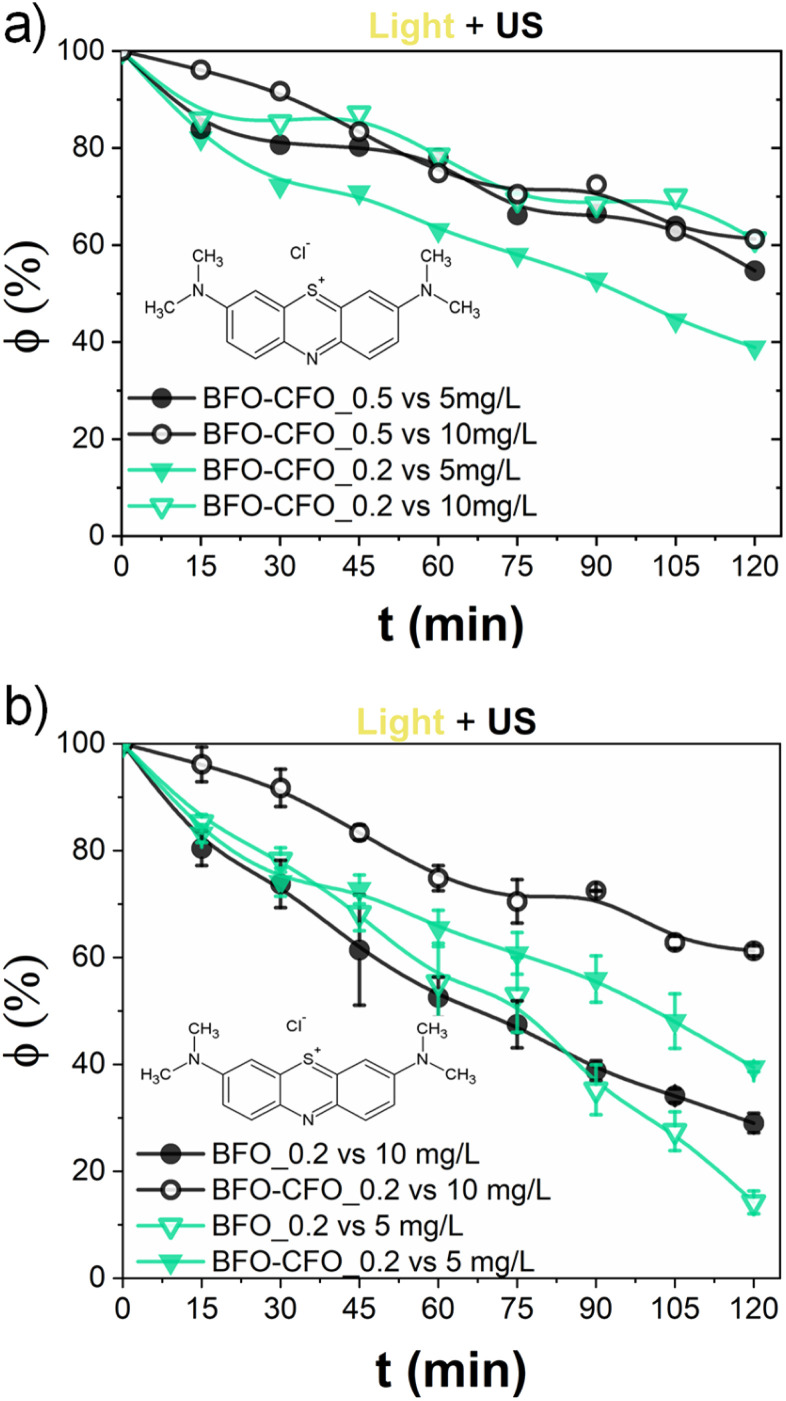
Residual methylene blue (MB) fraction (*φ*) after 120 min, obtained from piezo-photocatalytic (light on and ultrasounds) experiments with: (a) various loads of both hybrid catalyst and dye; (b) 0.2 g L^−1^ load of BFO and hybrid catalysts.

To further improve the catalytic performance and enable easy post-reaction recovery, a hybrid system combining BFO with CFO nanoparticles was developed. The resulting composite, denoted BFO–CFO (with 10 at% CFO), benefits from the ferrimagnetic nature of CFO, which allows magnetic separation—an advantage not afforded by antiferromagnetic BFO alone. Only piezo-photocatalytic experiments were conducted for these hybrids, shown in Fig. 5. The influence of catalyst concentration and dye loading was studied by testing both 0.5 g L^−1^ and 0.2 g L^−1^ of BFO–CFO in MB solutions of 5 and 10 mg L^−1^ (Fig. 5a). Lower catalyst concentrations led to improved light penetration and ultimately better degradation efficiency (*e.g.*, from ∼42 to 60% for 5 mg L^−1^ MB solutions). Applying this optimized loading strategy to pure BFO confirmed the trend; as reported in Fig. 5b, the combination of BFO at 0.2 g L^−1^ with 5 mg L^−1^ of MB yielded the highest catalytic activity, achieving nearly 90% degradation after 120 minutes (specifically 85.3% *vs.* 61.1% for BFO and BFO–CFO, respectively, against 5 ppm MB and 49.5% *vs.* 38.8% for BFO and BFO–CFO respectively, against 10 ppm MB).

**Fig. 6:**
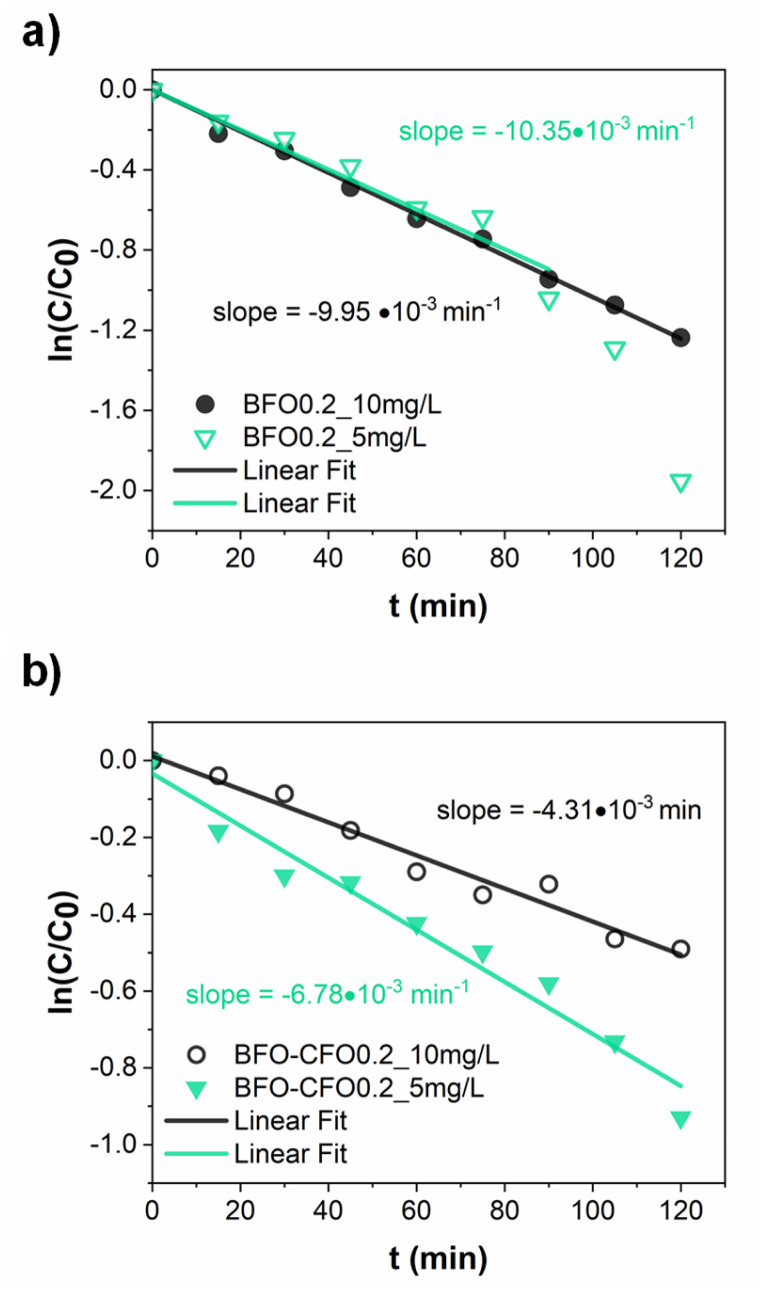
Data fitting of MB piezo-photocatalytic dissociation on (a) BFO and (b) BFO–CFO with a fist-order kinetics for two different initial concentration values *C*_0,1_ = 5 mg L^−1^and *C*_0,2_ = 10 mg L^−1^.

**Fig. 7:**
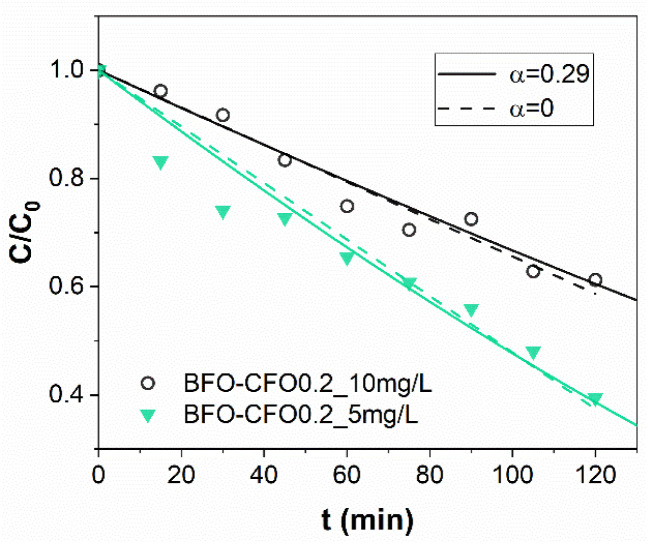
Data fitting of MB photodissociation on BFO–CFO with a zero-order and an α-order kinetics for two different initial concentration values *C*_0,1_ = 5 mg L^−1^ and *C*_0,2_ = 10 mg L^−1^.

To understand the kinetics of the process with these optimised operational parameters (catalyst loading, pollutant concentration), the piezo-photocatalytic degradation of MB was evaluated using BFO and BFO–CFO with fixed concentration (0.2 g L^−1^) at two initial dye concentrations (5 and 10 mg L^−1^). For the BFO catalyst, the experimental data aligned well with a pseudo-first-order kinetic model, as described by the Langmuir–Hinshelwood approach (see SI for more details) under conditions of low surface coverage,^68,69^ with a rate similar to values reported in literature.^8^ The similarity of the reaction slopes (*i.e.*, rate constants) for both concentrations in the early stages of degradation (Fig. 6a) supports this assumption, as the concentration ratio *C*/*C*_0_ should be independent on the initial conditions.^70^ At lower concentration (5 mg L^−1^), a slight acceleration of degradation was observed after 80 minutes, potentially due to reduced photon shielding in the increasingly transparent medium—a phenomenon also observed by Wang *et al.* in similar systems and ascribed to a two-stage pseudo-first-order kinetics.^71^ In contrast, the BFO–CFO system exhibited a concentration-dependent deviation from first-order behavior (Fig. 6b). The substantially different slopes at 5 and 10 mg L^−1^ suggested that surface adsorption and reaction dynamics were more complex in the composite. A subsequent fitting using zero-order kinetics failed to account for the scaling of degradation rates with concentration. Instead, a better correlation was obtained using a generalized power-law rate expression (shown in SI), yielding a fractional kinetic order *α* = 0.29 (Fig. 7). This departs from classical kinetic modeling of similar systems, which often assume constant reaction order regardless of surface composition or pollutant concentration.^72^ The sub-unity value of *α* implies that the reaction rate increases less than proportionally with MB concentration, suggesting the presence of competitive adsorption and partial site saturation. This behavior likely results from the complex surface environment of the hybrid catalyst: the introduction of CFO modifies the surface energy landscape, creating spatial heterogeneity and possibly introducing domains with lower activity or restricted accessibility. Such heterogeneity leads to the emergence of fractional-order kinetics, where the effective reaction rate depends not only on the concentration of MB but also on the distribution and availability of active sites.^73^ This interpretation is consistent with previous findings on mixed-phase systems and their influence on photocatalytic mechanisms.^74^ While our current analysis is limited to two dye concentrations at fixed catalyst loading, these findings already suggest that the kinetic regime is strongly influenced by both catalyst architecture and pollutant concentration. To further elucidate the origin of this fractional behavior and assess its generality, systematic studies involving different dye types, a broader range of concentrations, and varied catalyst loadings will be necessary. Furthermore, the observed kinetic behavior demonstrates that a magnetically recoverable catalyst system—achieved with only 10% CFO—can be optimized without compromising photocatalytic performance, offering a rational framework for designing multifunctional and reusable catalysts. To explore any subtle electronic interactions at the BFO–CFO interface, future studies will be carried out to assess the possible formation of heterojunctions and their impact on charge carrier dynamics.

### Magnetic recovery

**Fig. 8:**
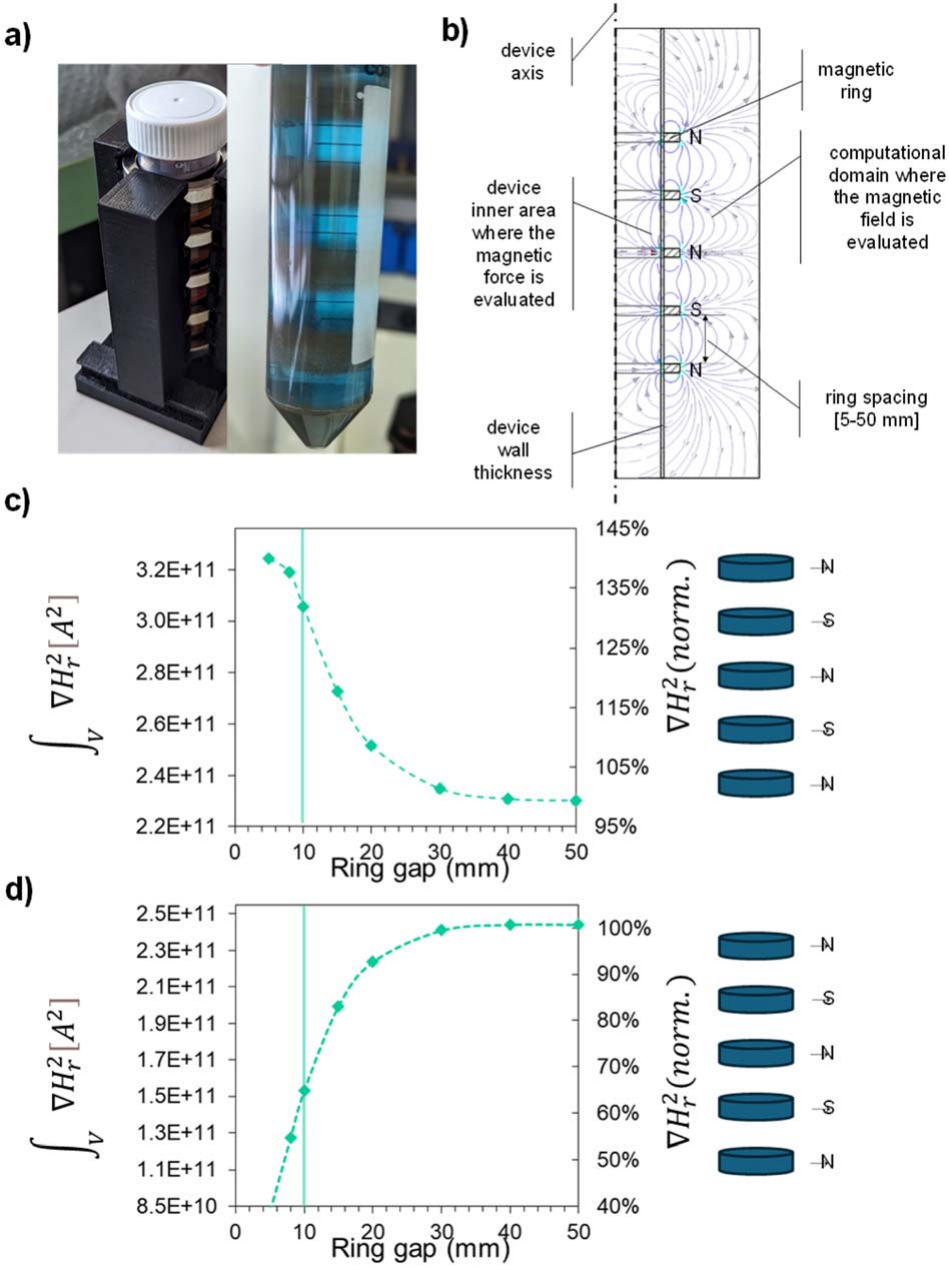
(a) Developed ring magnetic separator system, with the example of magnetic separation in the centrifuge tube; (b) axisymmetric 2D representation of the computational domain for the 5-ring system; (c and d) volumetric integral of the radial component of ∇Hr^2^ determining the total magnetic force *F*_M_ felt by the catalyst in the centrifuge section affected by the rings, in alternate and concordant configurations, respectively (values expressed as calculated, and as a percentage of the additive field value expected from the five magnets without interactions).

To enable efficient recovery and reuse of the BFO–CFO nanocomposite after water purification, a magnetic separation strategy was investigated by exploiting the intrinsic ferrimagnetic nature of CFO. A custom magnetic separator was developed using commercial ring permanent magnets (NdFeB, N35) to perform separation tests under static conditions. The separator (Fig. 8a and b) is equipped with 5-ring permanent magnets arranged to produce a high field gradient around a 50 mL centrifuge tube, containing the test sample. In a typical test, the catalyst was dispersed in a MB solution (5 mg L^−1^). To confirm that the container type did not influence the degradation efficiency, a control piezocatalytic experiment was performed using a 50 mL plastic centrifuge tube (the same vessel used for magnetic separation), yielding results comparable to those obtained in glass beakers. COMSOL simulations were performed to define the magnetic configuration maximizing the field gradient yielding the separation of the particles from the aqueous suspension. A parametric study was conducted by varying the mutual distance between the rings, specifically increasing it from 5 mm to 50 mm. The magnetic polarization of the rings (parallel to the tube axis) was tested in alternate and concordant configurations. The overall magnetic force was evaluated by integrating the radial component of the applied magnetic field square gradient (∇*H*^2^) across the device axis (Fig. 8b). ∇*H*^2^ determines the magnetic force (*F*_M_) exerted on the catalyst, defined by: *F*_M_ = *V*_NP_·*χ*_NP_·∇*H*^2^, where *V*_NP_ is the particles' volume and *χ*_NP_ their magnetic susceptibility.^75^Fig. 8c and d present the integrated values of the squared radial component of the magnetic field gradient 
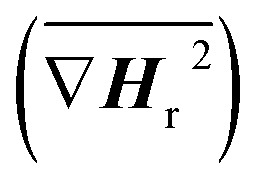
 as a function of the distance between ring magnets. The data reveal that magnetic fields generated by individual rings can interfere either constructively or destructively, depending on the spacing between them. By adjusting this distance, we identified configurations where the constructive interaction between the rings is maximized, resulting in enhanced magnetic forces. For the alternate configuration (Fig. 8c) the strongest magnetic interaction occurs at a 5 mm gap. Interestingly, even at a larger gap, where the radial magnetic force is reduced, the five-ring system still produces a field which remains higher than the simple additive contribution of the five magnets. At 10 mm, the estimated average squared radial field gradient is 
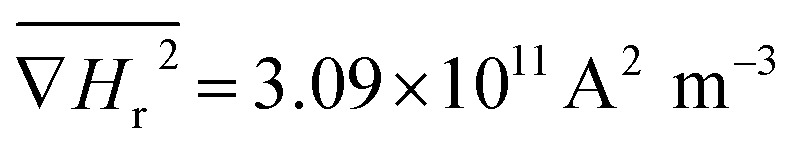
 (with a 32% gain compared to the additive contribution only), while decreases to 
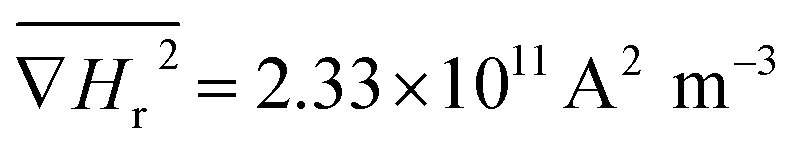
 at a 50 mm spacing. In the case of the concordant configuration (Fig. 8d), the device is expected to exert on the hybrid nanosystem the 
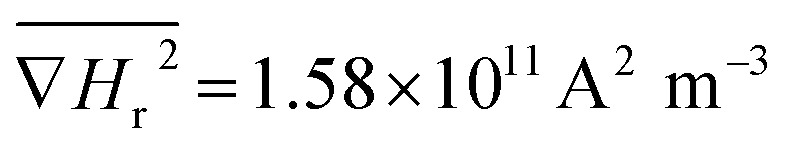
 at 10 mm (with a loss of 35% compared to the simple additive contribution) and increases to 
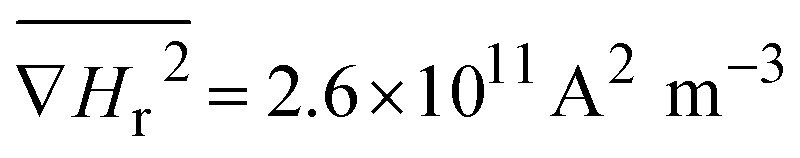
 at 50 mm. Therefore, at 10 mm, the alternate configuration allows a value 
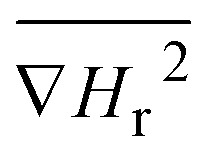
 to be obtained that is more than double that obtained from the concordant configuration (relative gain of 103%).

Based on our experimental results, the 10 mm gap in the alternate configuration was found to be the best choice to achieve the complete catalyst separation within a few seconds. This confirms that the chosen spacing provides an effective compromise between magnetic field strength and design feasibility for practical recovery devices. Notably, while both the ring-array setup and the single magnet achieved visually rapid aggregation (*i.e.*, within 2–3 seconds), only the 5-ring configuration ensured full recovery of the dispersed catalyst. Quantitatively, the ring setup achieved a 100% recovery yield, whereas the single-block magnet left a significant portion of magnetic nanocomposite suspended, yielding ∼20% recovery under the same conditions. This demonstrates that the enhanced magnetic gradient in the ring configuration is crucial not just for speed but for effectiveness—exerting a force strong enough to overcome Brownian motion and isolate even the smallest BFO–CFO hybrid particles from the aqueous phase, despite the low CFO content (10%), thus enabling reusability in subsequent catalytic cycles. These findings highlight the critical role of piezo-photocatalytic synergy in activating BFO-based systems and demonstrate the potential of magnetic nanocomposites like BFO–CFO for developing efficient, recoverable catalytic platforms.

## Conclusions

The photocatalytic performance of BFO was initially found to be limited under simulated solar light, with negligible degradation of organic dyes. However, when combined with ultrasonic vibration, BFO exhibited a clear enhancement in catalytic activity, especially under piezo-photocatalytic conditions. The photodissociation of methylene blue was significantly improved, likely due to better charge separation and reactive oxygen species generation induced by the piezoelectric effect. To improve recovery and usability, BFO–CFO nanocomposite was introduced, demonstrating promising piezo-photocatalytic performance alongside magnetic separability. These results suggest that combining piezoelectric-driven activation with magnetic recovery strategies is a viable route for developing efficient and recyclable catalytic systems.

## Experimental

### Chemicals

For the photocatalytic tests, methylene blue (MB) (Sigma Aldrich, Germany) and methyl orange (MO) (CARLO ERBA Reagents S.r.l., Cornaredo, Italy) were used as target pollutants. For the synthesis of the hybrid nanosystem, Bi(NO_3_)_3_·5H_2_O, Fe(NO_3_)_3_·9H_2_O, Co(NO_3_)_2_·6H_2_O and glycine (Sigma-Aldrich) were used.

### Experimental setup and conditions

#### Synthesis of BiFeO_3_ nanocrystallites

BiFeO_3_ (BFO) nanoparticles were synthesized through a glycine-assisted sol–gel combustion method.^47^ Bismuth and iron nitrates were dissolved in deionized water with a small amount of nitric acid to ensure complete dissolution, followed by the addition of glycine as a fuel and complexing agent. The mixture was stirred and gradually heated to promote gelation. Upon further heating, a self-sustained combustion reaction occurred, yielding a fluffy precursor powder. This powder was then subjected to thermal treatment in air at 350 °C for 1 h and subsequently at 500 °C for an additional hour to obtain phase pure crystalline BFO.

#### Synthesis of BFO–CFO hybrid nanosystem

For the synthesis of the BiFeO_3_–CoFe_2_O_4_ (BFO–CFO) nanocomposite, a similar combustion-based route was employed. Cobalt ferrite (CFO) nanoparticles were first prepared *via* a sol–gel self-combustion method using citric acid as a chelating agent, followed by gel formation and spontaneous combustion upon heating.^50,76,77^ These CFO seeds were then introduced into the BFO precursor solution prior to gelation, allowing their homogeneous incorporation within the matrix. The subsequent combustion and annealing steps mirrored those of the pure BFO synthesis, leading to the formation of CFO nanoparticles embedded within a crystalline BFO matrix.

#### Structural, morphological and magnetic characterizations

The powder samples were characterized using a Bruker D8 Advance diffractometer (solid state rapid LynxEye detector, Cu Kα radiation, Bragg–Brentano geometry, DIFFRACT plus software) in the 10–90° 2*θ* range with a step size of 0.013° (counting time was 4 s per step). The powder samples were grounded in an agate mortar and suspended in ethanol. A Si substrate was covered with several drops of the resulting suspension, leaving randomly oriented crystallites after drying.

A Philips CM200 transmission electron microscope (TEM), operating at 200 kV and equipped with a LaB_6_ filament, was used for the TEM analysis. Powdered samples of BFO or BFO–CFO were dispersed in ethanol and sonicated for one minute. A drop of the resulting suspension was deposited onto a commercially available holey carbon-coated TEM grid and allowed to air-dry until the ethanol had completely evaporated. The morphology and chemical composition of the BFO and BFO–CFO samples were examined using a Zeiss Supra 40 high-resolution field emission scanning electron microscope (FESEM) equipped with a Bruker Quanta 200 energy-dispersive X-ray spectroscopy (EDS) microanalyzer.

Magnetic field-dependent magnetization (M(H)) of the samples was collected using a PPMS magnetometer from Quantum Design Inc., at *T* = 10 K in the −5 T to +5 T field range and at *T* = 300 K in the −2 T to +2 T field.

#### Photocatalytic and piezo-photocatalytic experiments

Two different experimental setups were used. For the pure photocatalytic experiments, the solar radiation was simulated with one solar simulated light lamp (OSRAM ULTRA-Vitalux, 300 W) placed at 15 cm above the beaker, and the solution was kept under magnetic stirring. For the piezo-photocatalytic experiment the solar lamp was placed 15 cm above the beaker which was put in an ultrasonic bath (BANDELIN Sonorex, 35 kHz, 120 W). All experiments were conducted in an ice-cooled water bath, which effectively maintained the solution temperature below 30 °C throughout the experiment.

Experimental conditions are summarised in Table 1.

**Table 1:** Summary of conditions for performed experiments

Experimental conditions	
BFO-based materials concentrations	0.2 g L^−1^–0.5 g L^−1^
Pollutant concentration range	5 mg L^−1^–10 mg L^−1^
Experimental time	120 min
Temperature	25 ± 5 °C
Volume	50 mL

Quantitative measurements were performed with a Shimadzu UV-2600i UV-Vis spectrophotometer, monitoring the absorbance at wavelengths of 664.6 nm and 553.8 nm for MB and MO, respectively. The dye concentration was calculated according to a calibration line performed prior to each experiment, thanks to the Lambert–Beer law.

The experiments were monitored by taking aliquots at fixed times, every 15 minutes, and the residual dye fraction (*φ*) was calculated according to the following formula:
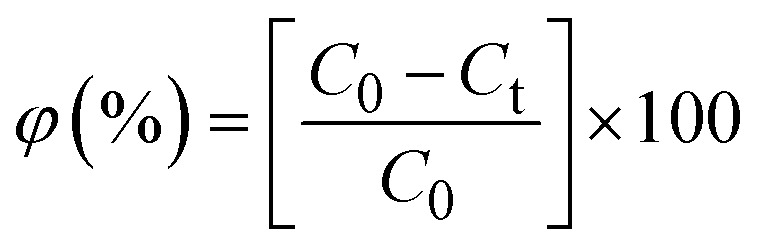
calculated by means of the initial concentration *C*_0_, corresponding to 5 or 10 mg L^−1^, and the concentration of the dye as a function of time *C*_t_.

#### Kinetic model

Kinetic modelling was conducted using BFO and BFO–CFO catalysts at two initial dye concentrations (5 and 10 mg mL^−1^). The system was approximated as pseudo-first-order, and data were fitted accordingly using:
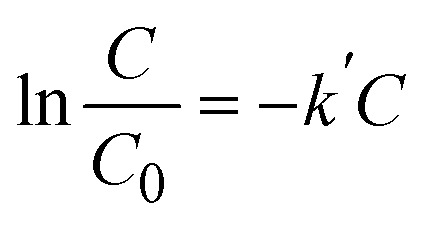
where *k*′ is a lumped kinetic constant (see SI for more details). The BFO–CFO composite exhibited significant deviations from first-order kinetics. As a result, a generalized power-law model^70^
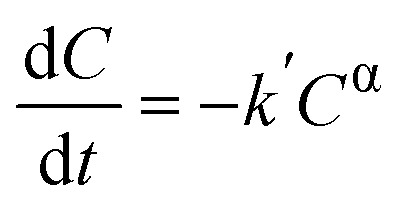
was adopted to better fit the experimental trends.

## Author contributions

P. M., N. G., S. A. and D. P. designed the experiments; P. M. and D. P. coordinated the data analysis and discussion. P. M. synthesized the nanoparticles and characterized them by XRPD and magnetometry techniques. G. B. and A. K. performed compositional, structural, and morphological characterization by EDS/SEM/TEM. M. V. performed simulations of the set-up of magnetic recovery. A. R. analysed the kinetic model for the systems. G. V. contributed to the discussion of the results. F. L., S. S., M. F., T. S. contributed to the results and discussion, and all the authors to the revision of the article. D. P. and M. F. acquired the funding and supervised the whole activity.

## Conflicts of interest

There are no conflicts to declare.

## Supplementary Material

NA-007-D5NA00646E-s001

## Data Availability

The datasets generated and/or analysed during the current study are available from the corresponding author on reasonable request. Supplementary information: (1) morphology and elemental composition, (2) photocatalytic properties of BFO *vs.* MB, (3) estimation of band-gap energy, (4) photocatalytic and piezo-photocatalytic properties of BFO *vs.* MO. (5) kinetics theory. See DOI: https://doi.org/10.1039/d5na00646e.
