# Supplementary material for: Insights into the piezo-photocatalytic activity and optimized magnetic recovery of hybrid bismuth ferrite-based nanosystems
Source: Nanoscale Adv. 2025 Aug 26;7(20):6551–62. doi: 10.1039/d5na00646e (PMC12409665; doi:10.1039/d5na00646e)
Supplement: NA-007-D5NA00646E-s001 [file NA-007-D5NA00646E-s001.pdf]

Supplementary Information (SI) for Nanoscale Advances.
This journal is © The Royal Society of Chemistry 2025

Supplementary Information

Insights into the Piezo-photocatalytic Activity and Magnetic Recovery of Hybrid Bismuth Ferrite-based Nanosystems

P. Maltoni*[a,b], N. Ghibaudo[a], A. Kumar[c], G. Barucca[c], M. Vocciante[a], F. Locardi[a], G. Varvaro[b], S. Slimani[a,b], M. Ferretti[a], T. Sarkar[d], A. Reverberi[a], S. Alberti*[a], D. Peddis[a,b]

Contents

S1. Morphology and Elemental Composition 2

S2. Photocatalyitic properties of BFO vs. MB 4

S3. Estimation of band-gap energy 4

S4. Photocatalyitic and piezo-photocatalytic properties of BFO vs. MO 5

S5. Kinetics theory 5

References 8

S1. Morphology and Elemental Composition

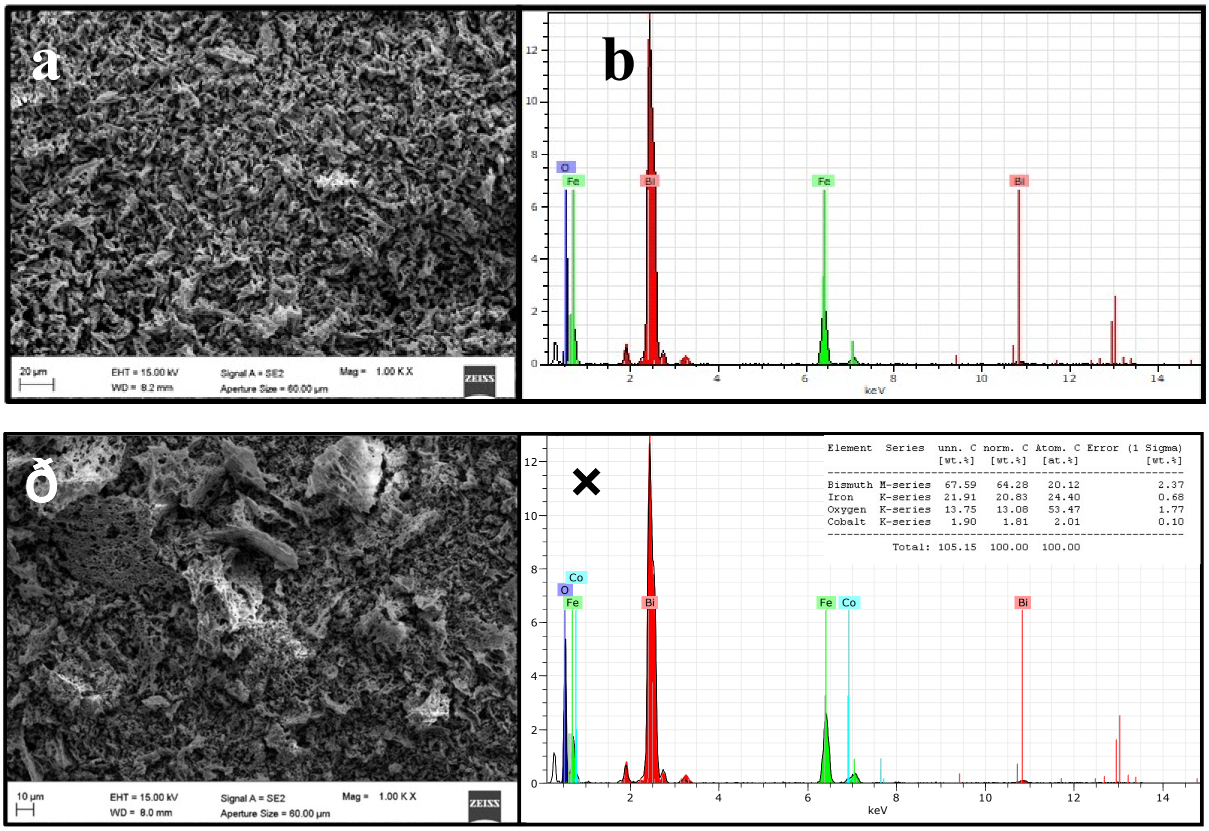

Figure S1. *BFO*: a) SEM (scanning electron microscopy) image showing the porosity of the sample; b) EDX spectrum and corresponding elements' percentages. The Bi atomic percentage is equal to the Fe percentage, as theoretically expected by the stoichiometry of $BiFeO_3$. The O percentage is smaller, but EDX technique is unsuitable for quantification of light elements such as O, so it should not be considered. *BFO-CFO composite*: c) SEM image showing the large porosity of the sample; d) EDX spectrum and corresponding elements' percentages. The Co atomic percentage is 10% of the Bi percentage, as theoretically expected by the stoichiometry and composition of the composite ($BiFeO_3$ (90%)-$CoFe_2O_4$ (10%)).

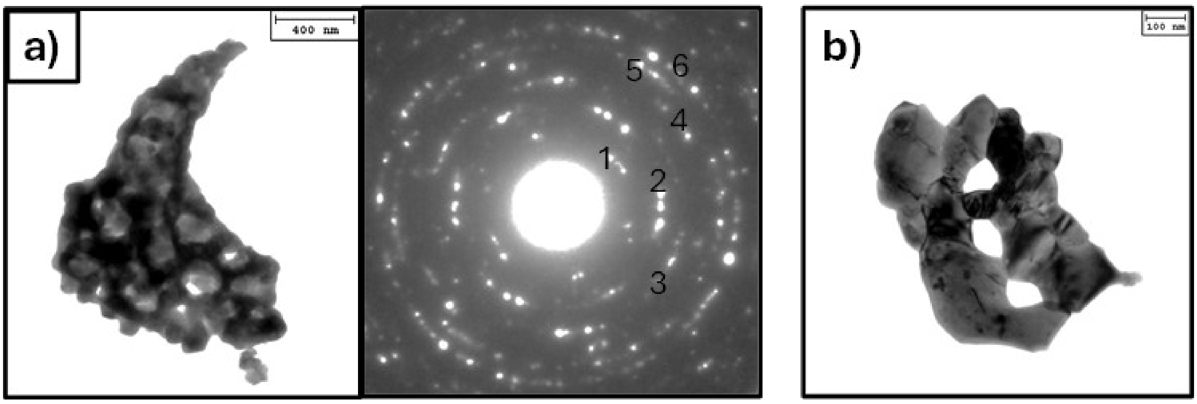

Figure S2. *Bright field TEM images of BFO*: a) general view showing the morphology of the sample and corresponding selected area electron diffraction (SAED) pattern; b) magnified image revealing that the sample is composed of aggregated irregular particles. All the interplanar distances associated with the diffraction spots visible in the SAED patterns are ascribable to the rhombohedral $BiFeO_3$ phase (International Centre for Diffraction Data (ICDD) card n° 71-2494). In particular, the spots belonging to the discontinuous diffraction rings and numbered from 1 to 6 correspond to the following interplanar distances: D1 = 0.395 nm; D2 = 0.278 nm; D3 = 0.227 nm; D4 = 0.195 nm; D5 = 0.175 nm; D6 = 0. 161 nm. These distances can be associated with: BFO(012) = 0.3968 nm; BFO(110) = 0.2793 nm; BFO(202) = 0.2284 nm; BFO(024) = 0.1984 nm; BFO(116) = 0.1780 nm; BFO(214) = 0.1617 nm.

S2. Photocatalyitic properties of BFO vs. MB

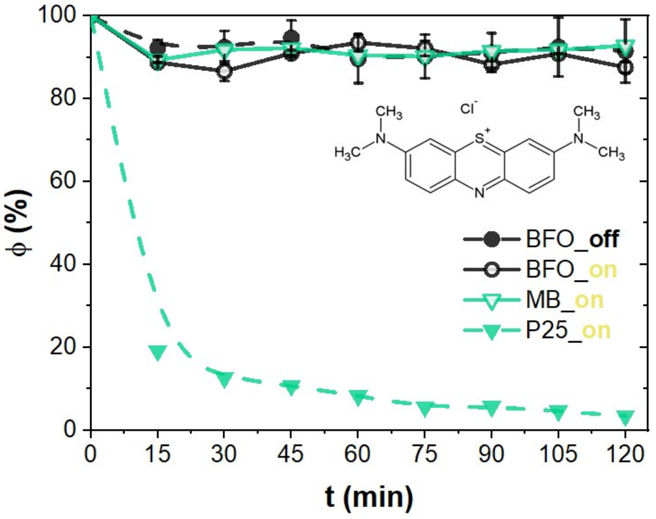

Figure S3. Residual methylene blue (MB) fraction (φ) after 120 min, obtained from photocatalytic (light on/off and catalyst) experiments.

S3. Estimation of band-gap energy

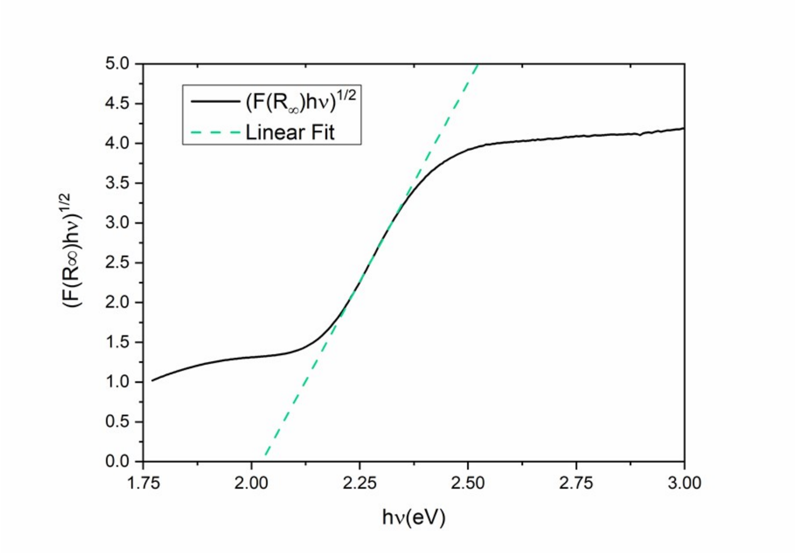

Figure S4. *Bandgap of BFO*: the band gap energy was estimated using absorption spectrum with the method proposed by Tauc[1]. The figure shows the diffuse reflectance spectrum of BFO after transformation following $(\alpha \cdot h\nu)^{1/\gamma}=B(h\nu-E_g)$, plotted against the photon energy. The region showing a steep, linear increase of light absorption with increasing energy is characteristic of semiconductor materials. The *x*-axis intersection point of the linear fit of the Tauc plot gives an estimate of the band gap energy.

S4. Photocatalyitic and piezo-photocatalytic properties of BFO vs. MO

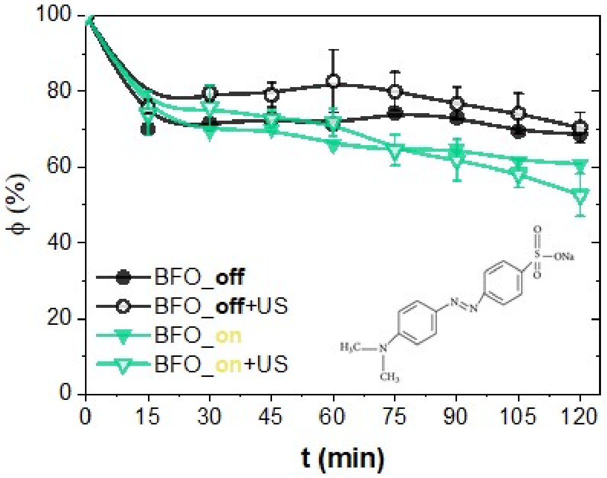

Figure S5. Residual methyl orange (MO) fraction (φ) after 120 min, obtained from photocatalytic and piezo-photocatalytic experiments.

S5. Kinetics theory

The Langmuir-Hinshelwood equation represents the generally adopted modelling to describe the concentration C of a reactant subject to photocatalytic degradation in a batch reactor[2], whose mass balance can be expressed as:

$$\frac{dC}{dt} = -r(C)\ (eq.1)$$

Where $r(C)$ is a kinetic expression depending on the role of both the solvent and the reaction intermediates, which may compete and interfere with the surface adsorption of the reactant on the active sites of the photocatalyst according to the expression[3]:

$$r(C) = \frac{k_r KC}{1 + KC + \sum_i K_i C_i}\ (eq.2)$$

Combining Eqs. (1) and (2) one obtains:

$$\frac{dC}{dt} = -\frac{k_r KC}{1 + KC + \sum_i K_i C_i}\ (eq.3)$$

Where C_i are the surface concentration of the adsorbed intermediates, K_i their relevant adsorption coefficients and k_r is the apparent kinetic constant of the photocatalytic dissociation, depending on radiation flux, catalyst mass, catalyst porosity and on other variables specifically related to the type of reaction[4].

When $KC+\sum_i K_iC_i \ll 1$ or when $KC+\sum_i K_iC_i \approx const.$, Eq. (3) leads to a first-order reaction according to the well-known expression:

$$ln\frac{C}{C_0} = - k^{'}C \ (eq.4)$$

Where k' is a lumped kinetic constant, not necessarily equal to k_rK. In his seminal paper[5], Ollis et al. pointed out that, in case of true first-order kinetics, the concentration ratio C/C_0 should be independent on the initial conditions, as predicted by Eq. (4).

When $KC \gg 1+\sum_i K_iC_i$, Eq.(3) gives a zero-order kinetics of the type:

$$\frac{C}{C_0} = 1 - \frac{k_r}{C_0}t \ (eq.5)$$

In this case, unlike the first-order kinetics described by eq.4, the slope of the interpolating line depends on the initial concentration value C_0. In many situations offered by real cases, the photodissociation kinetics belong to an intermediate order in a range [0,1].

With the above considerations in mind, the concentration data reported in Fig. 5b (see main text) pertaining to MB dissociation by BFO have been fitted by a first-order kinetic model, according to Eq.(4). The results are visualized in Fig. 6a (see main text), where the slopes corresponding to two different concentration values do not differ significantly from one another for early times. This result is suggestive of a first-order kinetics with good approximation, but the trend of MB dissociation on BFO at the lowest initial concentration value (5 ppm) requires a little consideration apart. In fact, while a linear trend is kept up to 80 min, a increasing degradation velocity can be observed for longer times. This phenomenon could be ascribed to an increased photodissociation yield, owing to a less photon shielding by the clear solution for longer times. An analogous trend was reported by Wang et al.[6], who observed a two-stage pseudo first-order kinetics in a photodissociation of methyl orange on Fe-doped and undoped TiO_2.

In Fig. 6b (see main text), The data pertaining to MB dissociation on BFO-CFO have been reported in semilogarithmic scale and a fit according to first-order kinetics was tested in analogy to Fig. 6a. In this case, however, the slope of the two fitting lines differ significantly, suggesting a considerable deviation from a first-order kinetics. For this reason, these data have been further interpolated in Fig. 7 (see main text) using both a zero-order and an intermediate α-order kinetics model, according to the following expression:

$$\frac{dC}{dt} = - k'C^{\alpha} \ (eq.6)$$

Whose integration leads to:

$$\frac{C}{C_0} = (1 - at)^b \ (eq.7)$$

with $a = \frac{k'(1 - \alpha)}{C_0^{1 - \alpha}}$ and $b = \frac{1}{1 - \alpha}$.

For a zero-order kinetics, namely for α = 0, one gets:

$$\frac{C}{C_0} = 1 - \frac{k'}{C_0} t \ (eq.8)$$

The two scatter plots of Fig. 7, referred to two different values of C_0, namely $C_{0,1}$ =5 ppm and $C_{0,2}$ = 10 ppm, have been preliminarly linearly interpolated in order to check the validity of Eq.(8). In case of zero-order kinetics, the slopes of the two curves $k'/C_{0,1}$ and $k'/C_{0,2}$ should scale as:

$$\frac{k'/C_{0,1}}{k'/C_{0,2}} = \frac{C_{0,2}}{C_{0,1}} \ (eq.9)$$

Linear data interpolation gave slope ratio equal to 1.517, poorly consistent with $C_{0,2}/C_{0,1}$=2, thus suggesting a rejection of a zero-order kinetics.

For this reason, a final data fitting according to Eq. (7) was tested. Assuming the independence of k' and α from C_0, one can observe that the two values of a, namely a_1 and a_2, each obtained by data regression from the respective curves of Fig. 7 should scale as:

$$\frac{a_1}{a_2} = \left(\frac{C_{0,2}}{C_{0,1}}\right)^{1 - \alpha} \ (eq.10)$$

In Fig. 7, the dashed curves represent data fitting for α=0.29, with a_1=4.0895·$10^{-3}$and a_2=2.5·10^{-3} for $C_{0,1}$=5 ppm and $C_{0,2}$=10 ppm, respectively. The goodness of fit for a zero-order and an α-order kinetics are essentially equivalent, but the latter case ensures the consistency with the experimental values $C_{0,2}/C_{0,1}$=2.

Hence, one can conclude that MB degradation by BFO-CFO follows an α-order kinetics with α=0.29.

References

1 J. Tauc, R. Grigorovici and A. Vancu, *physica status solidi (b)*, 1966, **15**, 627–637.

2 H. D. Tran, D. Q. Nguyen, P. T. Do and U. N. P. Tran, *RSC Adv*, 2023, **13**, 16915–16925.

3 D. Ollis, C. G. Silva and J. Faria, *Catal Today*, 2015, **240**, 80–85.

4 A. Fernández, G. Lassaletta, V. M. Jiménez, A. Justo, A. R. González-Elipe, J.-M. Herrmann, H. Tahiri and Y. Ait-Ichou, *Appl Catal B*, 1995, **7**, 49–63.

5 D. F. Ollis, *Front Chem*, DOI:10.3389/fchem.2018.00378.

6 X. H. Wang, J.-G. Li, H. Kamiyama, Y. Moriyoshi and T. Ishigaki, *J Phys Chem B*, 2006, **110**, 6804–6809.
